# Imaging the PM/AICD patient; a neuro-cardiac efficacy study

**DOI:** 10.1186/1532-429X-15-S1-P275

**Published:** 2013-01-30

**Authors:** June Yamrozik, Mark Doyle, Ronald B Williams, Sahadev T Reddy, Geetha Rayarao, Diane A Vido, Moneal Shah, Robert W Biederman

**Affiliations:** 1 Cardiac MRI, Allegheny General Hospital, Pittsburgh, PA, USA

## Background

Imaging patients with a pacemaker (PM) or AICD has been mostly taboo in the MRI environment. However, with current improvements in PMlead and generator development along with very vigilant and knowledgeable personnel in pacemaker safety, MRI procedures can be implemented successfully. However, safe performance, not withstanding the risks, leads one to question if the results from the scans provide additional valuable clinical information to warrant such risk.

Hypothesis: We propose that MRI imaging patients with a pacemaker can be crucial to establish clinical diagnosis.

## Methods

A total of 25 patients were imaged on a GE CV/i Excite Version 12, 1.5 T system (GE, Milwaukee, WI). Three patients had an AICD, 4 patients had an AICD/PM 2 pts had a single PM lead and the remaining 16 pts had a dual-chamber PM implantation. Each pt was performed in the dedicated Cardiac MRI Imaging Suite under the strict supervision of the Cardiologist. EP Lab personnel were present and reconfigured the PMor AICD into an appropriate asynchronous mode and/or with therapies under the guidance of the Cardiologist. The MRI scan sequences were selected such that the SAR level <2.0 W/kg.

## Results

All patients completed the procedure with no adverse events and the pacemaker was interrogated after the procedure by EP Lab. Impedance, thresholds, amplitudes and shock impedances were unchanged pre to post scanning. The average MRI scan time was 20±55min. Regarding the population, of the 25 patients imaged, 17 (68%) were neurology cases and 8(32%) were cardiac cases. After reviewing the results from the 17 neurology cases and comparing the results from prior studies (CT, angio and/or myelogram) 14/17 (82%) pts benefited from having this procedure. Additionally, 12/17(70%) patients altered the diagnosis resulting in improved outcome in patient care. The remaining 3(18%) pts MRI did not provide additional information that enhanced the diagnosis. The 8 cardiac cases were also compared to prior studies (heart cath, TEE, TTE and stress) and the outcome of 8/8 (100%) pts' diagnosis was shown to be enhanced by the CMR imaging. In 4/8(50%) of the 8 pts, CMR altered the prevailing clinical diagnosis while dramatically improving pt care. Thus, a total of 22 patients (88%) benefited by enhancement or alteration of the original diagnosis while 3(12%) patients did not provide any additional information.

## Conclusions

The use of PM/AICD imaging in MRI remains controversial but as the lead/generator technology has improved, increased confidence in its use is found. Herein, we show that MRI/CMR procedures on carefully selected patients with pacemakers/AICD's are beneficial and substantially enhance or alter patient diagnosis and downstream therapy. We propose that not only are PM/AICD's no longer taboo in the MRI environment but in the proper setting they can be markedly efficient with life-altering and life-saving consequences.

## Funding

None

